# A mixed method study on the impact of COVID-19 on mental healthcare in Ghana: rethinking mental health service delivery

**DOI:** 10.1186/s12939-024-02138-y

**Published:** 2024-03-14

**Authors:** Michael Zobi, Seth Kofi Abrokwa, Eugene Dordoye, Angel Phuti

**Affiliations:** 1 Institute of International Health, Global Health Centre, Charité Universitätsmedizin Berlin, 13353 Berlin, Germany; 2 Psychological Medicine & Mental Health Department, School of Medicine, University of Health and Allied Sciences, Hohoe, Ghana

**Keywords:** COVID-19, Mental healthcare, Mental healthcare workers, Ghana

## Abstract

**Background:**

Since its emergence, Severe Acute Respiratory Syndrome Coronavirus 2 (SARS-CoV-2) has caused severe health, social and economic challenges. Mental healthcare has been significantly affected globally, and even worse in developing countries. An emerging economy like Ghana in West Africa was not spared its disruptive effects. This study aimed to elucidate the impact of the coronavirus disease 2019, the COVID-19 pandemic (caused by SARS-CoV-2), on Ghana’s mental healthcare system.

**Methods:**

This is a mixed-method study using an emergent sequential exploratory design. A total of 15 front-line healthcare professionals were recruited from the three psychiatric hospitals, including the mental health department of a new teaching hospital in Ghana. Purposive sampling techniques and a semi-structured interview approach were used for recruitment and data collection. Quantitative data from hospital registries were collected and analysed to triangulate qualitative findings.

**Results:**

Fifteen mental health workers were enrolled in the study. The mean age of participants was (34.47 ± 4.07) years, average work experience of (6.23 ± 3.64) years and the majority as males (60%). This study found an average decline of 23% in hospital attendance and a 35% decline in admissions in all four facilities compared to the previous year, 2019. The lived experiences shared by mental healthcare providers were grouped under 3 main themes: Adjustments to workplace regulations, accessibility to mental healthcare, and psychological wellbeing of mental healthcare workers. The fear of contracting SARS-CoV-2 among healthcare workers, medication shortages, and logistical challenges were also reported to affect Mental Health services during the pandemic.

**Conclusion:**

This study highlights the challenges in mental healthcare during the COVID-19 pandemic in Ghana. The experiences encountered present an opportunity to gain insights into future pandemic preparedness and establish a framework for optimal mental healthcare delivery in Ghana.

**Supplementary Information:**

The online version contains supplementary material available at 10.1186/s12939-024-02138-y.

## Introduction

Severe acute respiratory syndrome coronavirus 2 (SARS-CoV-2) has garnered significant global attention since January 2020 due to its rapid transmission. On March 11, 2020, the World Health Organization (WHO) declared SARS-CoV-2 infection a public health emergency of international concern (PHEIC) [1]. Many lives and livelihoods were lost due to its devastating effects. Families and communities experienced separation since the introduction of preventive measures such as physical distancing, closing educational institutions, advising against non-essential travel, and promoting remote work [2, 3].

The Coronavirus disease (COVID-19) pandemic has significantly impacted not only the physical health of the population but also every aspect of our daily lives [4, 5]. Amidst these health, social, and economic ramifications, mental health has been significantly affected [6]. A surge in anxiety has been observed among many individuals, while for others, COVID-19 has triggered or intensified more severe mental health issues [4–7]. In general, while there is an increasing demand for mental health support, there has been a significant disruption to mental health services [8]. This disruption was particularly pronounced during the initial stages of the pandemic when personnel and resources were frequently redirected to COVID-19 response efforts. Studies from Spain, Australia, UK, Romania and Italy on the impact of the COVID-19 outbreak on mental healthcare have shown a decrease in the utilisation of mental health services, especially during the COVID-19 lockdown with also decreasing admissions since the onset of the COVID-19 pandemic [9–16]. These trends were mostly attributed to the limited bed numbers, ward closures, and fears of spreading the virus [17]. Other countries also experienced many changes in policy, legislation, guidelines and practices such as introducing personal protective equipment (PPE) for staff, self-isolation, social distancing and increased video consultations [17, 18]. Transition wards or corridors were also instituted in some instances for patients to await test results before admission or after discharge [17]. A rapid review of the impact of COVID-19 on work and personal outcomes in mental healthcare workers found that many mental healthcare workers were significantly impacted by the pandemic through increased workload, constant changes in roles and reported increased indirect trauma and workplace violence. There were also reported symptoms of burnout, psychological distress and psychosocial challenges for both those working in inpatient settings and those working remotely [19]. To address the consequences of the pandemic on mental health, public awareness and the provision of adequate mental health services, particularly among vulnerable groups who may face challenges in accessing such services, became even more crucial [6].

Ghana, a lower middle-income country, reported its first two cases of SARS-CoV-2 on March 13, 2020, and has since experienced numerous confirmed cases of SARS-CoV-2 and COVID-19-related deaths [20, 21]. In Ghana, an alarming 10% of the population suffer from mental illnesses, with a treatment gap of 98% [22–24]. This is primarily due to the over-reliance on institutionalised care for mental healthcare services [22]. In addition to this, the system suffers many deficiencies such as poor funding for mental health services, lack of human resources as well as inadequate in-patient and outpatient facilities [23, 25, 26]. Given the devastating impact of COVID-19 on the general healthcare system, it was imperative to investigate the specific impact of COVID-19 on a system with significant deficiencies even before the onset of the pandemic. Therefore, this study aimed to investigate and understand the impact of the COVID-19 pandemic on Ghana’s mental healthcare system.

## Method

### Study design

Using an emergent sequential exploratory design, we conducted a mixed-method study in four healthcare facilities in Ghana. Thus, we used quantitative data to triangulate key findings from the qualitative interviews [27]. This allows a more complete and synergistic use of data than the separate use of the various research methods [28, 29].

### Setting

There are only three psychiatric hospitals in Ghana and this research was conducted in all these hospitals, namely Accra Psychiatric Hospital, Pantang Hospital, and Ankaful Psychiatric Hospital. Additionally, we included the mental health department of Ho Teaching Hospital.

**Fig. 1:**
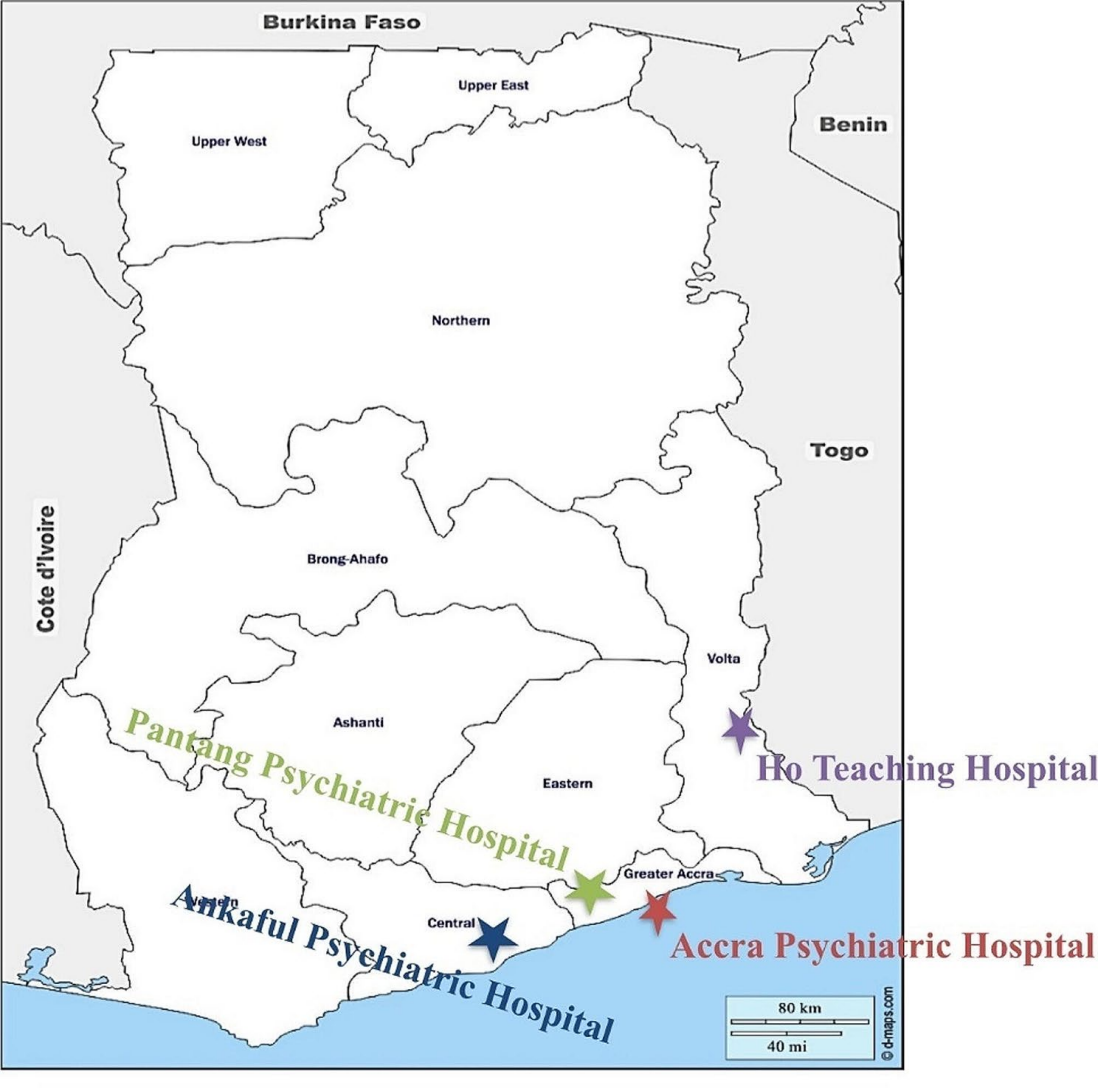
Map of Ghana showing locations of various mental health facilities that were included in evaluating the impact of COVID-19 on mental health service delivery in Ghana

The Accra Psychiatric Hospital is the first psychiatric hospital in Ghana located in the capital, Accra, with an academic affiliation with the University of Ghana School of Medicine and Dentistry for undergraduate training in Psychiatry and postgraduate training under the West African College of Psychiatrists (WACP) [30]. The Ankaful Psychiatric Hospital, located in Ankaful in the Komenda, Edina, Eguafo, Abrem Municipal Assembly of the central region of Ghana, was established to decongest the Accra Psychiatric Hospital in 1965 [31]. It serves healthcare seekers from the central, western, and Ashanti regions. The Pantang Hospital, like the Ankaful Psychiatric Hospital, was commissioned in 1975 to further decongest Accra Psychiatric Hospital. The hospital is situated near a village called Pantang, about 20 km from Accra, in the Ga East Municipal Assembly of the Greater Accra region of Ghana [32]. These three psychiatric hospitals provide mental healthcare services that include treatment, welfare, training, and rehabilitation. Ho Teaching Hospital is the fifth public Teaching Hospital in the country. It was re-commissioned by the health minister on 29th April 2019 after accreditation as a teaching hospital. It is in Ho, the capital city of the Volta region of Ghana, and provides specialised healthcare services including mental healthcare services, to the people of the Volta Region and other neighbouring countries, including the Republic of Togo, Benin, and the Federal Republic of Nigeria. Figure 1 shows the Ghana map locating the healthcare facilities where this study was conducted [33].

### Sampling strategy for the semi-structured interview

We enrolled participants using a purposive sampling strategy. The sample frame was defined as those front-line healthcare professionals, such as physicians, nurses, psychologists, and pharmacists, who offer mental health services in the facilities. Mental healthcare workers who had been working at the same mental health facility for at least six months before the outbreak of the COVID-19 pandemic were eligible to participate in the study. We excluded participants who neither gave consent nor signed the consent forms.

All three psychiatric hospitals in Ghana were selected for this study. Being a new teaching hospital with expected state-of-the-art facilities, the mental health unit of Ho Teaching Hospital was purposively selected in addition to the psychiatric hospitals. Other teaching and regional hospitals could not be interviewed due to time and financial constraints.

### Data collection

The data collection took place between July 2021 and August 2021. Due to COVID-19 restrictions, and after discussion with participants, we opted to collect qualitative data using Zoom online interviews. A total of 15 participants were recruited for semi-structured interviews. The phenomenological approach was used to conceptualise the interview guide, presented as supplementary material (Appendix 1). The first part of the interview guide comprises pre-interview demographic questions reporting the age, profession, and gender of participants. Major headings of interest were discussed with the study supervisor (AP) and open-ended questions were developed based on these headings. The questions consisted of an opening of broad descriptive questions, follow-up questions, probing questions on the domains of interest as well as eliciting recommendations from participants. Interviews were conducted by the lead researcher (M.Z). All interviews were conducted in English and were recorded. Pseudonyms were given to participants to ensure their anonymity. The interviewer had no prior relationship with the participants. To ensure the credibility and dependability of the study, the interviewer took notes on the participants’ body language and facial expressions and compared them with the themes that were obtained from the transcripts. Participants were encouraged to clarify concepts and validate ideas that were unclear to the researcher. By the 12th participant, data saturation was reached and three more participants were interviewed to consolidate the existing themes. Quantitative data were secondary data obtained from the hospital registries. The data collected included bed capacity, number of beds designated for COVID-19 case management, number of healthcare workers, annual hospital attendance and admissions between 2017 and 2022.

### Data analyses

We conducted descriptive statistics using Microsoft excel [34] for quantitative data analysis. The outcome of the analysis was summarised using frequency tables, graphs, means, and standard deviations.

For qualitative data analysis, audio-recorded interviews were transcribed verbatim, merged where appropriate with handwritten notes and coded. Audio recordings of interviews were replayed several times, cross-checking with transcripts and correcting all errors in the transcripts. Transcripts were de-identified by removing names and locations and checked for accuracy before being transferred to NVivo software (release 1.5) [35] for analysis. Coding was performed by MZ and AP and inter-coder reliability was conducted and discussed with the research team. Various details of the interviews were grouped under themes derived from the transcripts as agreed by all authors. Thematic analysis was used to analyse the data from the interviews. Themes were also discussed, and all disagreements were systematically resolved. Themes and sub-themes are presented with illustrative quotes, with participant pseudonyms.

## Results

### Characteristics of study participants

**Fig. 2:**
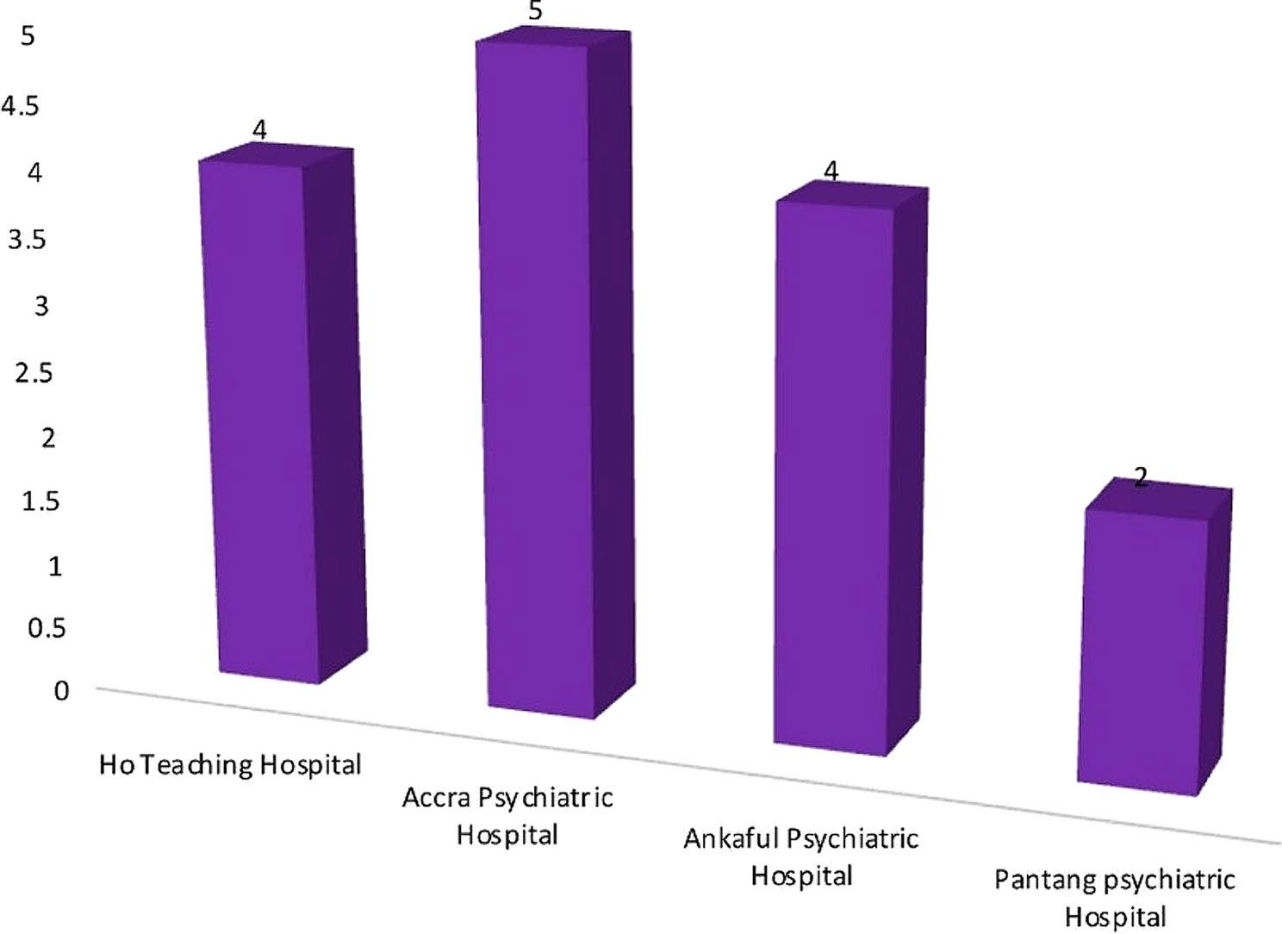
Distribution of participants of the semi-structured interviews according to the respective facilities where they work

**Fig. 3:**
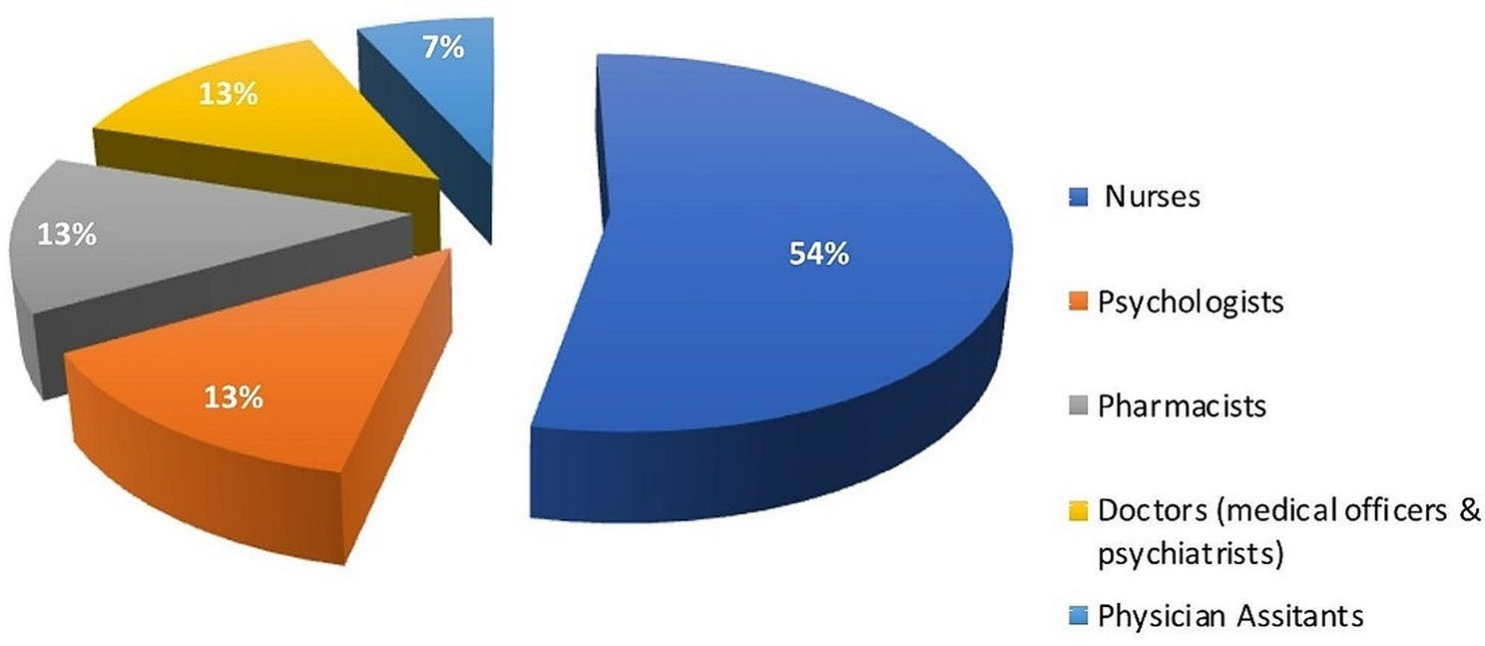
The distribution of different categories of healthcare workers sampled for the semi-structured interview on the impact of COVID-19 on mental health services delivery in Ghana

Fifteen (15) mental healthcare workers partook in the interview. These included mental health nurses, clinical psychologists, physician assistants, medical officers, psychiatrists, and pharmacists. The mean age of participants was (34.47 ± 4.07) years. The distribution of study participants by facility is shown in Fig. 2 below. About 60% of the participants were males. The mean work experience was (6.23 ± 3.64) years. Figure 3 shows the distribution of study participants by profession.

Perspectives of mental healthcare providers are grouped under 3 main themes: experiences related to workplace regulations, experiences related to care delivery, and consequences of some experiences. Where applicable, these findings were supported by secondary data from the hospital registries.

## Adjustments to workplace regulations

### Updated hygiene protocols and regulations

A two-metre gap was observed between healthcare workers and the patients in the consulting rooms. Unlike before the pandemic, when relatives of patients were allowed in the consulting rooms, the maximum number of people in a consulting room at a given time was reduced to three; comprising the patient, an attending clinician, and a nurse.

*In our consulting rooms, we have changed the position of the clients to maintain a two-metre distance between us. Also, we do not allow a lot of people into the consulting rooms; a maximum of three people in the consulting rooms”* (AE0006, Psychiatrist).

Five participants spoke about new hygiene practices that were incorporated into their facility in correspondence with the country’s new hygiene standards. For example, all persons visiting the health facilities were mandated to wear facemasks, have their temperatures checked, and wash their hands at the entrance of the facilities. Healthcare providers were also mandated use to face masks, face shields and scrubs instead of their usual uniforms during duty hours.

After COVID-19 testing became available in Ghana, patients were required to present a negative test result before an outpatient visit or for admission. It was mentioned that COVID-19 testing was expensive and thereby posing financial challenges to some patients.

*When COVID started, the test was a little expensive. They were paying [the patients] an amount that was even higher than what we would have taken for admission”* (LS0007, Medical officer).

### New regulation to reduce admissions

Bed capacity varied across the different facilities. Accra Psychiatric Hospital had a capacity of 394 beds, while both Pantang Psychiatric Hospital and Ankaful Psychiatric Hospital had capacities of 200 beds each. The mental health department of Ho Teaching Hospital, on the other hand, had a capacity of 10 beds.

Participants reported that they were allowed only 25% admissions of their maximum bed capacity. In some facilities, a new holding area was created, where patients awaited their COVID-19 test results before admission to the observation wards. Some participants also alluded that only critical cases were admitted at their facility.

*Yes, with respect to admissions, the capacity of the wards was quartered so they were taking a quarter of their maximum capacity. So, a ward for instance that was taking 10 patients was now taking at least 2 or 3 patients”* (AE0006, Psychiatrist).

**Fig. 4:**
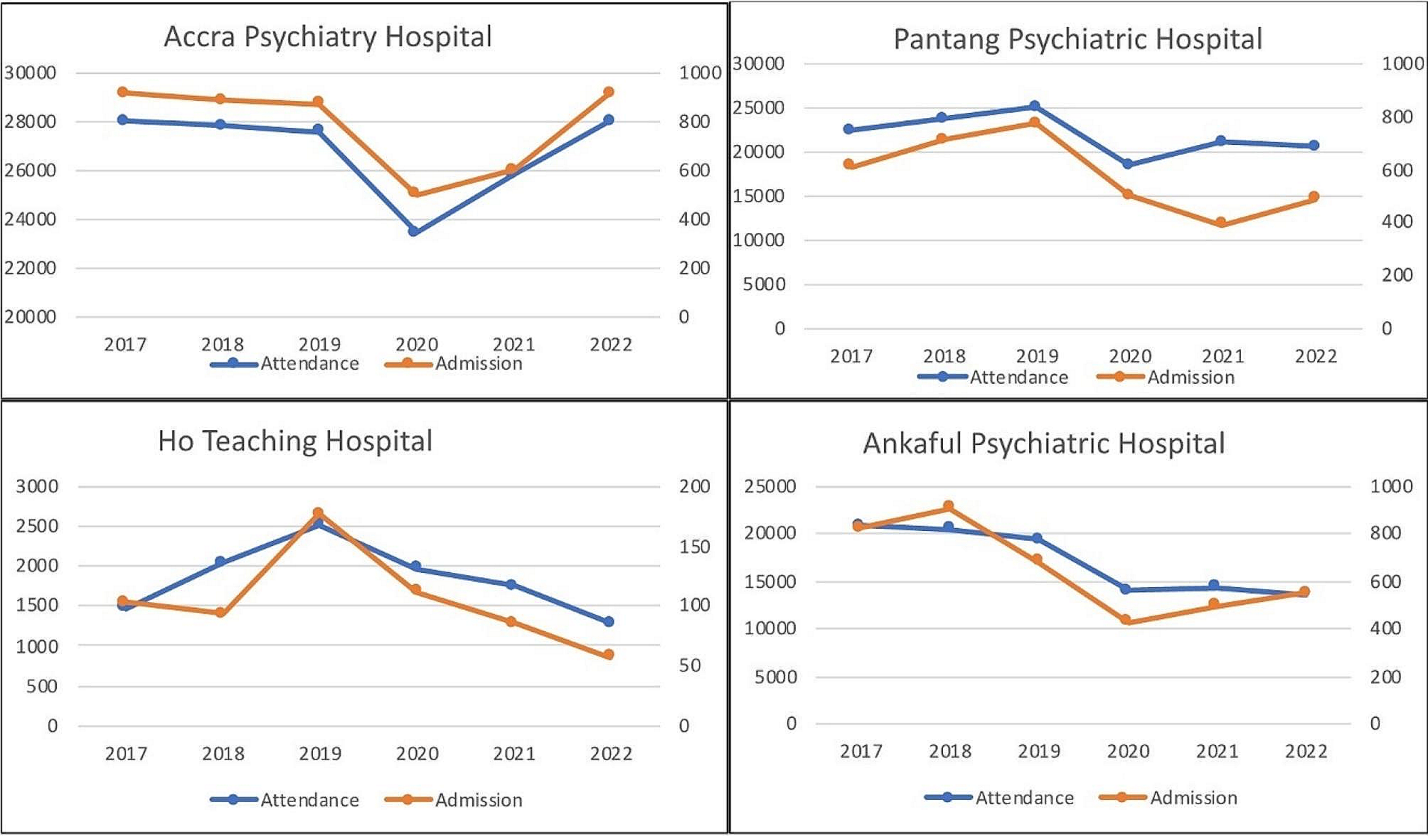
Trends in hospital attendance and admissions from 2017 to 2022 at Accra Psychiatric Hospital, Pantang Psychiatric Hospital, Ankaful Psychiatric Hospital and the mental health department of Ho Teaching Hospital

Corroborating the reports from study participants, we observed a decline in admissions in all four facilities from the onset of COVID-19 as shown in Fig. 4. When comparing admissions in 2019 and 2020, the percentage reduction in admissions at the Accra psychiatric hospital was 42%. Ankaful Psychiatric Hospital experienced a 37% decline, while Pantang Psychiatric Hospital and Ho Teaching Hospital observed a 35% and 24% decrease respectively.

## Accessibility to mental healthcare

### Scarcity of medical supplies

Among the participants, five individuals highlighted the scarcity of medical supplies such as personal protective equipment, and medications as a significant challenge encountered in the delivery of mental health services.

In addition to the existing inadequate logistics for daily operations before the onset of the pandemic, participants noted that insufficient personal protective equipment (PPE) like face masks, face shields, and gloves led mental healthcare workers to limit their interactions with patients due to concerns about contracting COVID-19. Mental healthcare workers who provide home visits expressed that sustaining such services became arduous, while those operating in ward settings were unable to have contact with patients unless properly attired in personal protective equipment. Also, as a result of insufficient personal protective equipment (PPE), certain healthcare workers contracted COVID-19.

*Care providers needed PPEs to be able to work because everyone was exposed so it tends to even cause a lot of stress to healthcare providers because there was agitation….…. We couldn’t get those PPEs on time and due to that, some [healthcare workers] were even exposed to the disease, and even the treatment took time for them to be able to recover”* CC0010, Pharmacist)

Furthermore, some participants highlighted that the pharmacies faced significant challenges due to the scarcity of medicines. This shortage arose from an insufficient supply of medications to mental health units, further exacerbated by border closures.

*…one of the challenges we faced in the pharmacy was the availability of drugs. The borders were closed, and you know in Ghana we only have three psychiatric hospitals as you are aware so definitely the drugs are not readily available”* (ESS0014, Pharmacist).

Some participants mentioned that the shortage of drugs in their facility challenged them to reach out to many suppliers of psychotropic medications, leading to the establishment of new professional alliances.

*So, we had two or three suppliers that if not for this pandemic I don’t think we would have even met them”* (ESS0014, Pharmacist).

### Reduced contact with patients and loss to follow-up

Five participants underscored that the services provided to patients when compared to before the onset of the pandemic are subpar. This was mainly because healthcare workers spent less time with patients, lack of active monitoring and follow-up, and delays in reviewing patients for feedback as there was frequent rescheduling of appointments.

*I was scared to see the patient, so as much as possible, I was spending less time with them in the consulting room… And you know how mental healthcare delivery is; the more you spend time, the more you develop rapport and the more you are able to make a good diagnosis.”* (AE0006, Psychiatrist).

*We only follow up through phone calls which some you will call and the number doesn’t even go through, some you will call and the number doesn’t even exist but those days [before COVID] after [calls], we also follow up to their homes but because of the pandemic we are not able to do that.”* (LO0008, Mental health nurse).

According to some participants, they had to reduce the duration and frequency of face-to-face contact with patients, especially when dispensing medication. For this reason, instructions on medication use and information on adverse effects were handed to patients in a written format. Some facilities made available phone numbers for further consultations when necessary. In some facilities, the pharmacy extended the allowable three-month Take-home doses of patient’s medication under the national health insurance scheme to five months. This initiative was to prevent patients from frequenting the hospital.

*Yeah, before COVID started we were giving [medications] for three months and that’s what the insurance allows. But during the pandemic, you know, we want to restrict patients coming into the hospital more often. With the fear that they may, or they might contract [COVID-19] so we decided that; okay fine, as a department, we can go an extra mile to give an extra two months so making a way for five months, especially for some of the psychotropics”* (ESS0014, Pharmacist).

These measures also saw the effect of decreased hospital attendance, which is buttressed by quantitative facility data. Comparing attendance in 2019 to 2020, Accra Psychiatric Hospital experienced a 15% decline in attendance. Similarly, Ankaful Psychiatric Hospital, Pantang Psychiatric Hospital and the mental health department of Ho Teaching Hospital reported a decrease of 27%, 26% and 22% respectively (Fig. 4).

### Discrimination in access to care

Three respondents highlighted stigma as a significant obstacle leading to the marginalisation of emergency services for persons with mental health conditions. Ghana typically witnesses extensive stigmatisation surrounding mental health, and the advent of COVID-19 exacerbated this issue with certain participants disclosing that individuals with mental health conditions and positive for COVID-19 encountered *“double stigmatisation* “, as expressed by one participant.

*… it was really disheartening because you call them* [the ambulance] *and they will be like “but this person has a psychiatric….”. It all comes down to people appreciating psychiatric conditions and the stigma that is attached to them. It makes it really hard because already there is stigma with mental illness and then a stigma with COVID. So, it’s like you are essentially dealing with a****double stigma****you know.”* (LS0007, Medical officer).

Facility data showed that at the onset of the COVID-19 pandemic, Accra Psychiatric Hospital designated 10 beds for COVID-19 case management. Pantang Psychiatric Hospital and Ho Teaching Hospital designated 18 beds and 2 beds respectively. However, it was reported that patients from psychiatric hospitals who were referred for specialized care at the national COVID-19 treatment centres were sometimes denied admission due to stigma. Some participants added that the ambulance services sometimes refused to transport these patients to the treatment centres. In such situations, the psychiatric hospitals had to manage the patients although they were not adequately resourced to manage physical illnesses.

*… we have to call the national ambulance to come to take [COVID-19] positive patients away. Immediately they hear****“oh it’s from…. psychiatric”***, *they place the psychiatric condition, which is not an emergency over the medical condition which is an emergency. So, we had a few cases like that, and they [the national ambulance] refused to come. They were saying we should manage the case ourselves. Just because the person has a mental health condition, our facility was left to manage our own cases.”* (LS0007, Medical officer).

### Consequential increase in relapses

According to five participants, there was a significant increase in the number of relapsed patients. This increase was attributed to reduced admissions, poor health-seeking behaviour during lockdown periods and reduced contact with patients such as the suspension of home visits and follow-ups. Additionally, participants mentioned that financial constraints faced by patients and their families played a role in treatment defaults and subsequent relapses.

*““In terms of our cases, we had a rise in both new and relapse cases. Relapse in the sense that clients were not comfortable coming to the hospital because they were scared of getting infected with the COVID so most of them were not coming. And some also because of financial challenges were not able to come to get their medications”* (OA0005, Mental health nurse)”.

## Psychological wellbeing of mental healthcare workers

### Feelings of fear and anxiety

Participants identified the fear of contracting the SARS-CoV-2 infection as another significant factor impacting the provision of mental health services. This was primarily due to several reasons: frequent infections among their colleagues, a lack of testing opportunities for both staff and patients, scarcity of medical supplies and an inadequate working environment that made it challenging to maintain safe physical distancing during triage and treatment. These circumstances resulted in heightened levels of anxiety among healthcare workers.

*…So, you feel that everybody is a suspect of COVID so getting closer to the patient, that kind of fear to even engage him/her in therapy or checking of vital signs. That fear was there so you are hesitant to perform such procedures for them.”* (JT0001, Mental health nurse).

Two participants shared that amidst the heightened fear of contracting COVID-19, they took responsibility to make the best out of the situation and hence being more cautious in adhering to hygiene protocols.

*…we can’t just hold on to the bad, you must make something good out of the bad. So, we made our way through, yes. By sensitising yourself that you have to protect yourself more before the patient.”* (DAD0015, Mental health nurse).

### Complains of increased workplace stress

In all four facilities, nurses constituted the majority of the workforce. Accra Psychiatric Hospital had 448 nurses, 6 psychiatrists, 2 psychologists, and 17 physician assistants. Pantang Psychiatric Hospital had 335 nurses, 8 psychiatrists, and 2 psychologists. Ho Teaching Hospital had 36 nurses, 3 psychiatrists, and 2 psychologists for their mental health department. Ankaful Psychiatric Hospital had 242 nurses, 2 psychiatrists, and no psychologist. The average psychiatrist-to-nurse ratio was 1:80 in the 3 main psychiatric hospitals and 1:12 in the Ho Teaching hospital. The average psychologist-to-nurse ratio was 1:195 in the 3 main psychiatric hospitals and 1:13 in the Ho Teaching hospital.

To reduce overcrowding with a consequent reduction in the potential of the spread of COVID-19, facilities re-grouped healthcare workers, mainly nurses, into smaller teams for daily shifts. Additionally, participants underscored that infection among staff led to a decrease in the workforce as the infected individuals were required to isolate themselves. These changes resulted in additional stress due to increased workloads and longer working hours. It was emphasised that when a team member was absent, the remaining team had to shoulder extra responsibilities leading to even higher levels of stress.

## Discussion

The COVID-19 pandemic had far-reaching effects on mental health services in Ghana, necessitating significant adaptations to protocols and service delivery. In this study, we examined the changes made in mental healthcare in Ghana since the onset of the pandemic, highlighting the challenges faced by mental healthcare workers and their impact on service delivery.

### Changes in mental health facility protocols

To contain the spread of SARS-CoV-2, mental health facilities in Ghana implemented various adaptations to their protocols. These included admitting only severe emergencies, utilising telemedical platforms for consultations, conducting COVID-19 screenings and tests before clinic visits, and extending prescription durations of medications. The reduction in admissions was drastic, with some facilities admitting only critical cases while others held patients in a designated area until a negative COVID-19 test result was obtained.

The use of stringent criteria for admissions was mainly due to the meagre number of beds in the psychiatric hospitals in the country, a concern that has been raised by several authors as well as the national report on the mental health system of Ghana [23, 26, 36].

A situational analysis revealed that Ghana’s three state-owned psychiatric hospitals provide just 7.04 beds per 100,000 population [36]. The average bed capacity in the main psychiatric hospitals was 265 beds, with Ho Teaching Hospital having only 10 beds. Consequently, the number of beds designated for COVID-19 case management was notably low, posing significant challenges during times of increased demand. Similar scarcities of psychiatric beds have been reported globally with severe ramifications on mental health services [37–39].

To curb the spread of COVID-19, facilities aimed to reduce overcrowding by instituting reservation systems for hospital visits, limitations on the number of people per consulting room, and the use of telemedicine platforms. Hospital attendance and admissions also declined due to lockdown measures, costly COVID-19 testing, and exacerbated poverty due to pandemic-related unemployment. Telehealth emerged as a viable alternative, with remote video or phone calls, online therapies, and digital applications being widely adopted [39, 40]. The global adoption of telemedicine has quickly surpassed traditional face-to-face care, demonstrating its immediate effectiveness owing to the high acceptance levels among patients [39, 41]. In the present study, some participants expressed concerns about the limited availability of telemedicine platforms for mental health care delivery. While psychiatrists and nurses did not specifically use telemedicine in their practices, psychologists used it complementarily for interventions such as psychotherapy. It is noteworthy that telemedicine is still a relatively new field in Ghana with ongoing pilot projects and plans for its integration into the conventional healthcare system [42].

COVID-19 protocols, including personal protective equipment, temperature checks, hand washing, and testing were integrated into service delivery protocols at all mental health facilities in this study. Similar measures have been observed in various parts of the world, with certain facilities incorporating staff screening for COVID-19 as part of their infection prevention protocol [38, 39]. The scarcity of protective equipment and the difficulty in explaining hygiene measures to patients posed significant challenges causing anxiety among the healthcare workers due to fear of contracting the disease and thereby hindering close contact with clients. Likewise, in other countries, the insufficient availability of personal protective equipment was recognized as causing anxiety among frontline healthcare workers [43]. Conversely, there was a rise in relapses among patients due to frequent appointment rescheduling, delayed or inadequate intervention, and insufficient therapy monitoring.

### Resource allocation challenges

The mental health system in Ghana suffers from poor resource allocation, leading to inadequate beds, irregular medicine supplies, unequal distribution of facilities across the country, and a lack of personal protective equipment during a pandemic [23–25]. Additionally, the concentration of psychiatric hospitals in the southern region makes mental health services inaccessible for individuals in other parts of the country, resulting in late presentations and worsened conditions [25]. Addressing resource allocation challenges and expanding access to mental health facilities beyond the southern belt are crucial steps toward ensuring comprehensive and equitable nationwide mental healthcare.

### Improving mental health service delivery in Ghana

This section highlights key recommendations from the study, focusing on the need for a nationwide upgrade and decentralisation of mental health services in Ghana. Additionally, it emphasises the importance of personnel training, improving work conditions for mental health service providers, health education, and adequate funding.

#### Scaling up mental health services

This study suggests a comprehensive approach to enhance mental health services in Ghana. It proposes incorporating mental health care into primary health care, enabling district hospitals and health centres to manage basic mental health conditions and emergencies. This aligns with the recommendations from the mental health system assessment report, which advocates for day-treatment centres in each region and accessible rehabilitation opportunities [25]. Shifting from traditional institutional service provision to community-based mental health services is also recommended, aiming to decentralise care and alleviate the burden on the three psychiatric hospitals in the country [25].

#### Personnel training and improving work conditions

Investing in personnel training and continuous professional education is crucial for improving mental health service delivery. The government could ensure the availability of free accredited refresher courses to keep mental healthcare workers updated with current practices. Moreover, increasing training opportunities for mental healthcare workers at all levels and providing incentives to attract professionals, such as risk allowances, could effectively address workforce shortages [25, 44].

#### Health education

Streamlined and targeted health education is vital in reducing stigma and promoting positive health-seeking behaviours among the public. Implementing legislation to protect individuals with mental health challenges regarding job security, accommodation, and access to holistic treatment is recommended. The study also proposes the promotion of advocacy groups to raise awareness and advocate for the rights of people with mental health problems [25].

#### Funding

Given the low government spending on mental health in low-income settings like Ghana, political will and increased funding are critical. The study emphasises the need for a clear delineation of funding plans for mental health. However, the government’s low prioritisation of mental health may hinder the implementation of these proposals [23].

### Limitations

Due to COVID-19 restrictions, this study was conducted online, which may result in excluding participants who lacked conditions for online interviews. However, the study’s findings align with existing literature on the impact of COVID-19 on mental health systems in other LMICs and publications regarding the state of Ghana’s mental health system. Only summary reports were obtained for the quantitative data therefore statistical inferences could not be made. Because conclusions cannot be drawn from these results in terms of statistical significance nor generalised to the entire country or other lower middle-income settings, countrywide and multi-country studies are recommended, including interventional research to expand mental health services in low- and middle-income settings. Additionally, further exploration of pandemic preparedness in mental healthcare is essential to equip this marginalised sector for future unforeseen circumstances.

## Conclusion

This study brings attention to the existing shortcomings in mental healthcare. It emphasises the influence of COVID-19, including the alterations in healthcare policies within various mental health facilities in Ghana in response to the pandemic. This paper underscores that Ghana’s mental healthcare system is not adequately resourced and requires strengthening in all aspects. The experiences encountered during the COVID-19 pandemic present an opportunity to gain insights into future pandemic preparedness and establish a framework for optimal mental healthcare delivery in Ghana. A crucial aspect of seizing this opportunity involves re-evaluating government expenditure on mental health and fostering favourable working conditions for mental healthcare workers.

To the best of our knowledge, this is the first research of its kind conducted in Ghana. Our study could therefore serve as a starting point for policymakers to review emergency preparedness and response strategies for mental health in Ghana. Preventive measures and mental health promotion strategies such as regular screening programs, accessible psychological educational resources, capacity building for community, crisis, and home-treatment teams as well as upscaling telemedicine services and integrating mental health services into existing primary care models could be feasible solutions.

### Electronic supplementary material

Below is the link to the electronic supplementary material.

Supplementary Material 1

## Data Availability

Recorded interviews cannot be available due to confidentiality and ethical considerations. However, transcripts are available from the corresponding author but not for public use as participants did not explicitly consent to share with third parties. Data may be available upon reasonable request and with the permission of the regulating ethics committee and participants.

## Abbreviations

COVID: 19 Coronavirus disease 2019
DAAD: Deutscher Akademischer Austauschdienst
LMIC: Lower middle-income country
PHEIC: Public Health Emergency of International Concern
PPE: Personal Protective Equipment
SARS-CoV-2: Severe Acute Respiratory Syndrome Coronavirus 2
WACP: West African College of Psychiatrists
WHO: World Health Organization
