# Supplementary material for: A mixed method study on the impact of COVID-19 on mental healthcare in Ghana: rethinking mental health service delivery
Source: Int J Equity Health. 2024 Mar 14;23:56. doi: 10.1186/s12939-024-02138-y (PMC10941419; doi:10.1186/s12939-024-02138-y)
Supplement: Supplementary file 1 — Supplementary Material 1 [file 12939_2024_2138_MOESM1_ESM.docx]

**The Impact of COVID-19 on mental healthcare in Ghana: rethinking mental health service delivery.**

**Supplement material**

Appendix I: Interview guide

Appendix I: Interview guide

Tell me more about yourself and your position in the hospital…………

1. Are there National protocols for mental healthcare delivery in Ghana?

- Yes
- No

1. If yes, are these national guidelines in use your health facility?

- Yes
- No
- Tell me more about them ___________________________________

1. Have there been changes made to these protocols to adjust for service delivery during the pandemic?

- Yes
- No

1. What changes were made? ………………………………………………………………………………….
2. How have these changes affected mental healthcare delivery? ……………….
3. Do you see more cases than you used to see before the pandemic? ……………….
4. Please list the most common conditions which you see since the beginning of the pandemic in to order of decreasing frequency.
5. What challenges have you faced in mental healthcare delivery during this pandemic?
6. How have these challenges you faced affected your mental healthcare delivery?
7. Which deficiencies in mental health system do you think contributed to these challenges in mental health care?
8. What are the possible ways of improving mental healthcare in Ghana?
